# *Neobacillus terrisolis* sp. nov. and *Neobacillus solisequens* sp. nov. Isolated from Soil

**DOI:** 10.3390/microorganisms13112437

**Published:** 2025-10-24

**Authors:** Haoyu Wu, Congguo Ran, Nan Zhou, Xize Zhao, Xingyu Liu, Chengying Jiang, Yinghao Zhao, Ying Lv

**Affiliations:** 1 State Key Laboratory of Geological Processes and Mineral Resources, China University of Geosciences, Beijing 100083, China; 2 State Key Laboratory of Microbial Diversity and Innovative Utilization, Environmental Microbiology Research Center, Institute of Microbiology, Chinese Academy of Sciences, Beijing 100101, China; 3 University of Chinese Academy of Sciences, Beijing 100049, China

**Keywords:** *Neobacillus terrisolis* sp. nov., *Neobacillus solisequens* sp. nov., *Bacillaceae*

## Abstract

Two bacterial strains, designated LXY-1^T^ and LXY-4^T^, were isolated from soil samples collected at a heavy metal smelting plant located in Guangxi, China. Phylogenetic analysis indicated that these strains formed two distinct lineages within the genus *Neobacillus*. Both strains were characterized as facultative anaerobic, Gram-positive staining, endospore-forming, non-motile, short-rod bacteria. The major cellular fatty acids identified in these strains included C_16:0_, iso-C_15:0_, antéiso-C_15:0,_ and antéiso-C_17:0_. The predominant polar lipids comprised diphosphatidylglycerol (DPG), phosphatidylethanolamine (PE), and phosphatidylglycerol (PG). The average nucleotide identity (ANI) values between the newly isolated strains and their closest phylogenetic relatives, the type strains of the genus *Neobacillus*, were found to be below 95%, with corresponding digital DNA–DNA hybridization (dDDH) values remaining below 70%. Based on a comprehensive polyphasic taxonomic analysis incorporating chemotaxonomic, phenotypic, phylogenetic, and genomic data, we proposed that strains LXY-1^T^ and LXY-4^T^ represent two novel species of the genus *Neobacillus*, for which the names *Neobacillus terrisolis* sp. nov. and *Neobacillus solisequens* sp. nov. are designated. The type strains are LXY-1^T^ (= CGMCC 30313^T^ = JCM 37671^T^) and LXY-4^T^ (= CGMCC 1.62901^T^ = JCM 37672^T^), respectively.

## 1. Introduction

Recently, Patel and Gupta proposed the establishment of the genus *Neobacillus* based primarily on whole-genome phylogenetic reconstruction and the identification of conserved signature indels (CSIs) as key phylogenomic markers [1]. At the time of writing, the formal classification of the genus *Neobacillus* includes 31 validly published names (https://lpsn.dsmz.de/search?word=Neobacillus, accessed on 2 September 2025); these 31 validly published species are reclassifications of species previously in *Bacillus*. Recent studies have demonstrated that members of this genus exhibit a broad ecological distribution [2,3,4,5], having been isolated from a diverse array of environments and hosts, including terrestrial soils [6], aquatic systems [7], roots [8], and rhizosphere environments [9,10]. Some species were isolated from specific habitats, such as deep sea sediment samples [11], compost material [12], and also human feces [13]. The two novel strains LXY-1^T^ and LXY-4^T^ (Appendix A) were isolated from soil in a heavy metal smelting plant. The soil surrounding heavy metal smelting plants is persistently contaminated with high concentrations of heavy metals and toxic compounds. Under such extreme environmental conditions, microbial strains capable of survival are likely to have developed specialized resistance mechanisms. These strains exhibit significant potential for bioremediation by reducing heavy metal and toxic substance levels through processes such as adsorption, transformation, or degradation, thus contributing to the restoration of polluted environments. Screening these strains not only provides valuable microbial resources for bioremediation applications and supports the ecological recovery of contaminated soils but also facilitates in-depth investigation of microbial resistance mechanisms, thereby advancing the field of environmental microbiology.

It is reported that the genus *Neobacillus* exhibits diverse functional roles, including the synthesis of silver oxide nanoparticles [14], as well as the prevention and treatment of plant blight [15]. Notably, in the sulfur cycle, the genus *Neobacillus* is involved in hydrogen sulfide degradation [16], sulfide reduction [17], and oxidizing sulfide and sulfane into sulfite [18]. The genus *Neobacillus* exhibits a close phylogenetic relationship with the genus *Bacillus*, the latter of which has been extensively studied for its role in heavy metal bioremediation, like that involved in the remediation of cadmium and nickel [19] and Cr (VI) and Pb (II) [20].

The strains LXY-1^T^ and LXY-4^T^ were characterized through a comprehensive analysis encompassing 16S ribosomal RNA (rRNA) gene sequencing [21], the profiling of fatty acid methyl ester compositions derived from whole-cell hydrolysates, as well as additional biochemical and physiological assessments. Furthermore, whole-genome sequencing was conducted to elucidate their genomic architectures, followed by comparative genomic analyses against the type strains of their most closely related species to investigate evolutionary relationships and genomic distinctiveness. Whole-genome sequencing and comparative genomic analysis provide unprecedented rigor in the classification of novel bacterial species, serving as a cornerstone of modern taxonomy. By resolving complete genomic sequences, these approaches enable precise determination of average nucleotide identity (ANI) and genomic homology, overcoming the limitations of traditional phenotypic or 16S rRNA gene-based methods and allowing for reliable differentiation of closely related species. Comparative genomics further reveals horizontal gene transfer events, core genome signatures, and divergences in metabolic pathways, providing objective, molecular-level evidence for species demarcation. This genome-based taxonomic framework substantially improves the resolution and reproducibility of bacterial classification.

In recent years, the demand for diverse precious metal resources in various aspects of human production and daily life has been steadily increasing. Progress in efficient, cost-effective metallurgical technologies depends on harnessing in situ native microbial cultivation. In this study, we report the isolation of two strains, LXY-1^T^ and LXY-4^T^, from a heavy metal smelting plant and propose their classification as two novel species within the genus *Neobacillus*: *Neobacillus terrisolis* sp. nov. and *Neobacillus solisequens* sp. nov. This taxonomic proposal is substantiated through comprehensive analyses of their genotypic, chemotaxonomic, and phenotypic characteristics. Our findings contribute to the expansion of the *Neobacillus* strain repository and provide both theoretical foundations and valuable microbial resources for improving the efficiency of polymetallic ore metallurgy.

## 2. Materials and Methods

### 2.1. Isolation and Culturing

Soil samples were collected from a heavy metal smelting plant in Guangxi, China (23°42′2″ N, 109°12′10″ E). Approximately 20 g of soil was aseptically collected in a centrifuge tube and transported to the laboratory at a temperature of 4 °C. In total, 5 g of soil sample and 45 mL of sterile water were added into one centrifuge tube, shaking vigorously for 10 min to ensure that the soil particles were fully suspended. The soil mixture was left to stand still for 10 min, then the solid particles were separated. The supernatant was the bacterial suspension used for screening. The resulting suspension was then serially diluted 10-fold, ranging from 10^−1^ to 10^−7^. Each dilution was spread onto R2A agar plates and incubated at 30 °C for 3 days. The selected medium demonstrates superior suitability for cultivating a wide phylogenetic range of environmental microorganisms, including those with slow growth rates and oligotrophic nutritional preferences [22].

A total of 50 clones were screened and purified using the streak-plate technique. Among these, strains LXY-1^T^ and LXY-4^T^ were identified as potential novel species through 16S rRNA gene sequence comparisons using the EzBioCloud database (https://www.ezbiocloud.net/, accessed on 2 September 2025) [23,24]. Strains LXY-1^T^ and LXY-4^T^ were stored at −80 °C as 50% (*v*/*v*) glycerol suspension. They were subsequently deposited in the China General Microbiological Culture Collection Centre (CGMCC) and the Japan Collection of Microorganisms (JCM) under the following accession numbers: LXY-1^T^ = CGMCC 30313^T^ = JCM 37671^T^ and LXY-4^T^ = CGMCC 1.62901^T^ = JCM 37672^T^.

### 2.2. Morphological and Physiological Analysis

Phylogenomic reconstruction based on IPGA [25] (https://nmdc.cn/ipga/, accessed on 2 September 2025) robustly placed strains LXY-1^T^ and LXY-4^T^ within the genus *Neobacillus*, with each strain forming a well-supported monophyletic clade.

Phenotypic characteristics, biochemical properties, polar lipids, and cellular fatty acids were analyzed under optimal growth conditions. Cell morphology was examined using scanning electron microscopy (SU8010, Hitachi, Tokyo, Japan), and cell motility was evaluated by light microscopy (Axiostar Plus, ZEISS, Oberkochen, Germany). Gram staining was carried out in accordance with the manufacturer’s protocol using a Gram staining kit (Catalog No. G1060, Solarbio, Beijing, China) [26]. To determine the temperature range supporting growth, strains were incubated at 4, 22, 30, 37, 42, 50, and 60 °C for 72 h. The pH range for growth was assessed at intervals of 1.0 pH units between pH 4.0 and 12.0, with incubation at 30 °C for 72 h. NaCl tolerance was evaluated in liquid R2A medium supplemented with 0–5% (*w*/*v*) NaCl at 1% increments. Cell growth was quantified by measuring the optical density at 600 nm (OD_600_) using a UV/Vis spectrophotometer (SPECORD 205; Analytik Jena, Jena, Germany). Biochemical and enzymatic characteristics were analyzed using GEN III microplates (Biolog, Hayward, CA, USA), the API 20 NE system, and the API ZYM system (bioMérieux, Marcy-l’Étoile, France), following the manufacturers’ instructions [27]. Antibiotic susceptibility was assessed using the single-disk diffusion method [28], with inhibition zone diameters recorded as indicators of sensitivity. Whole-cell fatty acid composition and polar lipid profiles were determined using previously established protocols [29].

The production of H_2_S resulted in the blackening of lead acetate, and the reaction product was detected using lead acetate test paper (Art. No. RZK01472). The pH value was measured using a pH meter (Mettler Toledo, Greifensee, Switzerland).

### 2.3. 16S rRNA Gene Sequencing and Phylogenetic Analysis

The 16S rRNA gene of the pure culture was amplified by using universal primers 27F (5′-AGAGTTTGATCCTGG CTCAG3′) and 1492R (5′-GGTTACCTTGTTACGACTT-3′), under the following conditions: 95 °C for 5 min; 30 cycles of 95 °C for 30 s, 55 °C for 30 s, 72 °C for 1.5 min; and final extension at 72 °C for 10 min [30]. The PCR products were sequenced by Beijing Tianyihuiyuan Biotechnology (Beijing, China) [31]. By comparing the 16S rRNA gene sequences with the bacterial strain types available in the EzBioCloud database (https://www.ezbiocloud.net/, accessed on 2 September 2025), the bacterial identification results were obtained. The phylogenetic trees were reconstructed using the neighbor-joining (NJ) method [32] according to Kimura’s two-parameter model [33] in MEGA (version 7.0), using the maximum-likelihood (ML) method [34] based on the Tamura–Nei model and the maximum-parsimony (MP) method [35]. Levels of support for the nodes were obtained with 1000 bootstrap replicates for NJ and ML and 500 for MP analysis (Figures S1 and S2) [36].

### 2.4. Genome Sequencing and Analysis

Genomic DNA extraction was performed following previously established protocols [37]. The genomic DNA library was constructed utilizing the Illumina TruSeq DNA Library Prep Kit. Specifically, genomic DNA was subjected to fragmentation to achieve a size range of 300–500 base pairs (bp) using a Covaris M220 focused ultrasonicator (Covaris, Inc., Woburn, MA, USA). Subsequently, the fragmented DNA underwent end-repair and was ligated with TruSeq adapters. Following size selection (targeting 350–550 bp fragments) and purification with AMPure XP beads (Beckman Coulter Life Sciences, Indianapolis, IN, USA), the library was denatured and subjected to sequencing on the Illumina HiSeq X Ten platform, generating 2 × 150 bp paired-end reads. Sequence quality control was implemented using fastp (v0.20.0), while gene and coding sequence (CDS) prediction were carried out employing Prodigal (v2.6.3) and GeneMarkS (v4.3), respectively. The raw sequence data were subjected to a comprehensive quality assessment [38], followed by trimming and de novo assembly using SOAPdenovo (v2.04) [39]. To assess the phylogenomic classification and evolutionary relationships among the strains, average nucleotide identity (ANI) and digital DNA–DNA hybridization (dDDH) values were computed between the bacterial strains under investigation and their phylogenetically closest relatives. dDDH values were determined via the web-based Genome-to-Genome Distance Calculator (GGDC, v2.1; http://ggdc.dsmz.de/ggdc.php, accessed on 2 September 2025) [40], while average nucleotide identity (ANI) was determined using the online ANI calculator (ANI Calculator|EzBioCloud.net) (https://www.ezbiocloud.net/tools/ani, accessed on 2 September 2025).

## 3. Results and Discussion

### 3.1. Morphological and Physiological Investigation

**Figure 1:**
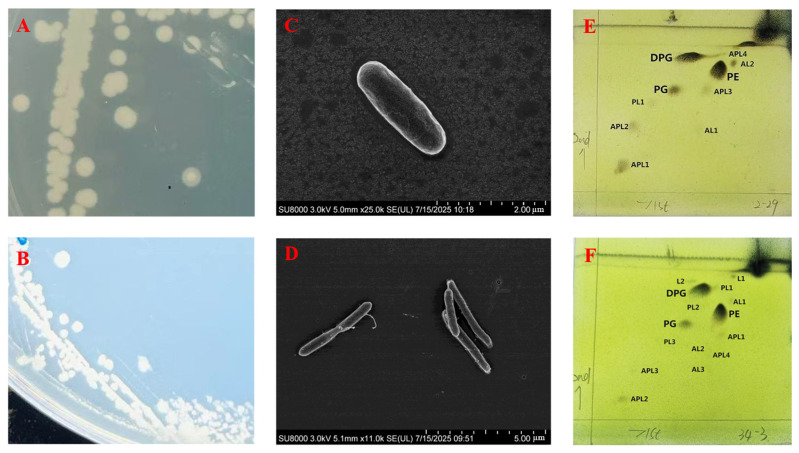
Cell and colony morphology of strains LXY-1^T^ and LXY-4^T^. (**A**,**B**) Colony morphology after three days of cultivation on R2A agar plates for strains LXY-1^T^ and LXY-4^T^; (**C**,**D**) scanning electron micrograph of LXY-1^T^ and LXY-4^T^; (**E**,**F**) polar lipids profiles of strains LXY-1^T^ and LXY-4^T^. DPG: diphosphatidylglycerol; PE: phosphatidylethanolamine; PG: phosphatidylglycerol; PL: phospholipid.

LXY-1^T^ formed round, creamy-white, 1–2 mm opaque colonies with moist (Figure 1A), slightly raised surfaces on R2A solid medium. Strain LXY-4^T^ formed round, 0.5–1 mm semi-transparent colonies with moist (Figure 1B), slightly raised surfaces on R2A solid medium. Scanning electron microscopy revealed that both strains were exhibited rod-shaped morphology (Figure 1C,D). Comparative analysis of cellular morphology highlighted distinct differences between strains LXY-1^T^ and the reference strain *Neobacillus drentensis* LMG 21831^T^. Specifically, strain LXY-1^T^ was characterized by short-oval shaped morphology, whereas strain *Neobacillus drentensis* LMG 21831^T^ demonstrated a tapered-rod shaped morphology. Strain LXY-4^T^ demonstrated a rod-shaped cellular morphology, in contrast to the slightly tapered rod-shaped morphology of *Neobacillus bataviensis* LMG 21833^T^. These morphological distinctions suggest potential taxonomic differentiation among these strains. Gram staining confirmed that strains LXY-1^T^ and LXY-4^T^ were Gram-positive.

The growth phenotypes of strains LXY-1^T^ and LXY-4^T^, including their responses to temperature, pH, and NaCl, were characterized as follows: both strains LXY-1^T^ and LXY-4^T^ could grow at temperatures from 22 to 50 °C and showed optimal growth at 22 °C, indicating possible physiological and ecological adaptations across these strains. Strain LXY-1^T^ could grow in the R2A medium at pH 6.0–12.0, while strain LXY-4^T^ could grow in the R2A medium at pH 7.0–12.0. The optimal pH for growth was 10.0 for both strains LXY-1^T^ and LXY-4^T^ (Table 1). NaCl tolerance tests revealed that strain LXY-1^T^ could grow in the presence of 0–4% (*w*/*v*) NaCl, with an optimal NaCl concentration of 0% (*w*/*v*). In contrast, strain LXY-4^T^ could grow in the range of 0–3% (*w*/*v*) NaCl, with an optimal NaCl concentration of 0% (*w*/*v*). In the experiment of producing H_2_S, both lead acetate test papers in strains LXY-1^T^ and LXY-4^T^ turned brown, showing that H_2_S was produced.

The substrate utilization capabilities of strains LXY-1^T^ and LXY-4^T^ were systematically analyzed using GEN III MicroPlates (Biolog) (Biolog, Hayward, CA, USA), API ZYM, and API 20 NE systems (bioMérieux, Marcy-l’Étoile, France). GEN III MicroPlate (Biolog, Hayward, CA, USA) results revealed significant differences in carbon source utilization compared with their respective type strains: LXY-1^T^ could metabolize common substrates including dextrin, N-acetyl-D-glucosamine, α-D-glucose, D-fructose, glycerol, D-glucose-6-PO_4_, D-fructose-6-PO_4_, D-serine, gelatin, L-alanine, L-serine, pectin, D-gluconic acid, methyl pyruvate, L-lactic acid, L-malic acid, bromo-succinic acid, and formic acid, which exhibited a broader substrate spectrum compared with *N. drentensis* LMG 21831^T^ [41]. Compared with *N. bataviensis* LMG 21833^T^ [41], LXY-4^T^ could metabolize gentiobiose, sucrose, *β*-methyl-D-glucoside, D-Salicin, N-acetyl-D-glucosamine, α-D-glucose, D-fructose, Inosine, D-sorbitol, D-mannitol, D-arabitol, glycerol, pectin, D-saccharic acid, D-Lactic acid methyl ester, L-lactic acid, L-malic acid, α-hydroxy-butyric acid, and acetic acid, while *N. bataviensis* LMG 21833^T^ lacked the ability to utilize D-arabitol (Table S1).

Enzymatic activity profiles analyzed by API ZYM and API 20 NE systems showed that LXY-1^T^ exhibited activities for alkaline phosphatase, esterase (C4), esterase (C8), aminopeptidase, trypsin, chymotrypsin, acid phosphatase, phosphoamidase, α-Glucosidase, *β*-glucosidase, N-acetyl-glucosamine, potassium gluconate, adipic acid, mannitol, maltose, malate, and trisodium citrate. Besides alkaline phosphatase, trypsin, acid phosphatase, phosphoamidase, *β*-glucosidase, N-acetyl-glucosamine, mannitol, potassium gluconate, and malate, LXY-4^T^ was also active for *β*-Glucosidase but was negative for esterase (C4), esterase (C8), aminopeptidase and chymotrypsin, glucose, maltose, adipic acid, and trisodium citrate. Similarly, both strains LXY-1^T^ and LXY-4^T^ were negative for lipase (C14), valine aminopeptidase, cysteine aminopeptidase, α-Galactosidase, *β*-Galactosidase, *β*-Glucuronidase, glucosaminidase, α-mannosidase and α-fucosidase, tryptophan, glucose, arginine dihydrolase, urease, arabinose, mannose, capric acid, phenylacetic acid, and cytochrome oxidase (Tables S2 and S3).

**Figure 2:**
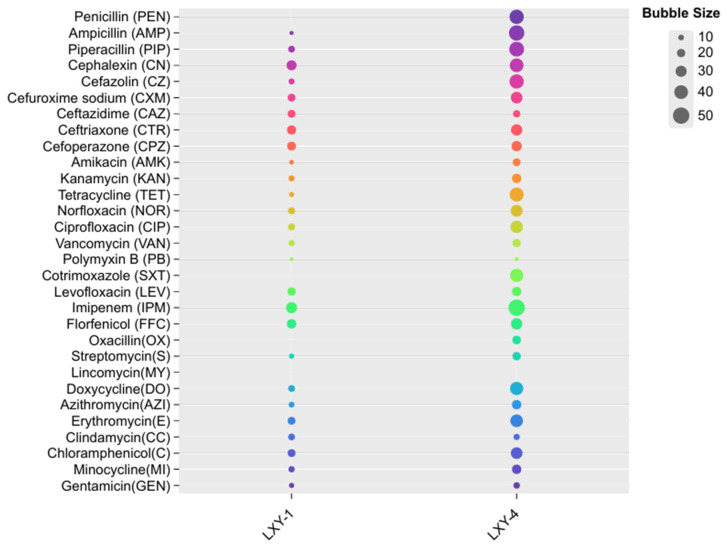
Antibiotic inhibition zones of strains LXY-1^T^ and LXY-4^T^. The bubble size indicates the size of the inhibition zone (mm).

The antibiotic susceptibility profiles of strains LXY-1^T^ and LXY-4^T^ revealed both shared and distinctive resistance patterns (Figure 2). Both strains exhibited marked resistance to *β*-lactam antibiotics, including cefazolin, cefuroxime sodium, ceftazidime, ceftriaxone, and cefoperazone and showed moderate sensitivity to aminoglycosides such as gentamicin, kanamycin, and streptomycin. However, LXY-1^T^ displayed higher sensitivity to clindamycin than LXY-4^T^. In contrast, LXY-4^T^ exhibited high sensitivity to penicillin, cotrimoxazole, and oxacillin, whereas LXY-1^T^ showed no sensitivity to these antibiotics. Both strains LXY-1^T^ and LXY-4^T^ were not sensitive to lincomycin. These phenotypic patterns serve as important taxonomic markers and may reflect distinct ecological adaptations associated with the resistance profiles of each strain.

### 3.2. Phylogenetic Analysis

**Figure 3:**
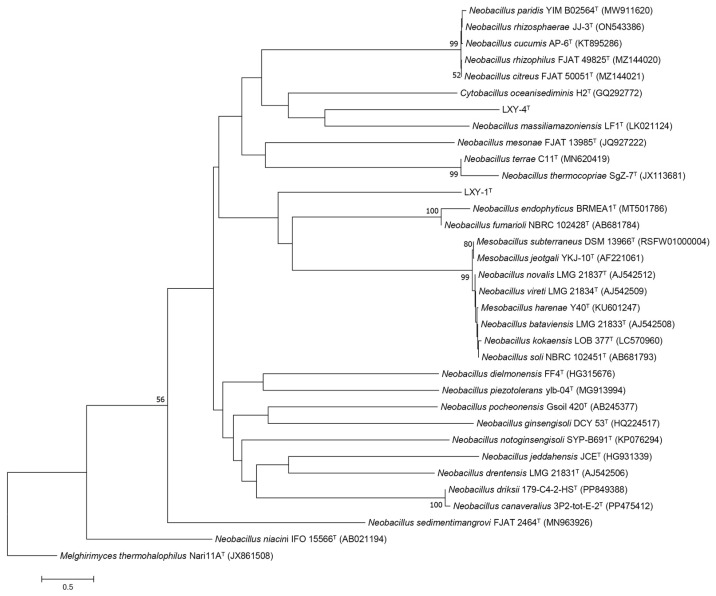
Phylogenetic tree of strains LXY-1^T^, LXY-4^T^, and their closely related type strains within the genus *Neobacillus*. The tree was constructed using the neighbor-joining method based on the 16S rRNA sequence. *Melghirimyces thermohalophilus* Nari11A^T^ served as the out-group. All sequences were retrieved from GenBank; accession numbers are provided in parentheses. Numbers at branch node indicate confidence levels represented by bootstrap support values (only values ≥ 50% are shown) based on 1000 replicates. The scale bar represents 0.5 substitutions per nucleotide position.

The 16S rRNA gene sequences of strains *Neobacillus terrisolis* sp. nov. and *Neobacillus solisequens* sp. nov. are PP800207 and PQ578319, respectively. Analysis of 16S rRNA gene sequences indicated that the closest relative of strain LXY-1^T^ was the type strain *Neobacillus drentensis* LMG 21831^T^ (with 98.27% 16S rRNA gene sequence identity) [41], followed by *Neobacillus cucumis* AP-6^T^ (98.16%) [9] and *Neobacillus rhizosphaerae* JJ-3^T^ (98.10%) [10] (Figure 3). For strain LXY-4^T^, the closest relative was the type strain *Neobacillus bataviensis* LMG 21833^T^ (97.40%) [41], followed by *Mesobacillus subterraneus* DSM 13966^T^ (97.40%) [42] and *Neobacillus soli* NBRC 102451^T^ (97.33%) [41] (Figure 3).

### 3.3. Genome Characteristics

**Figure 4:**
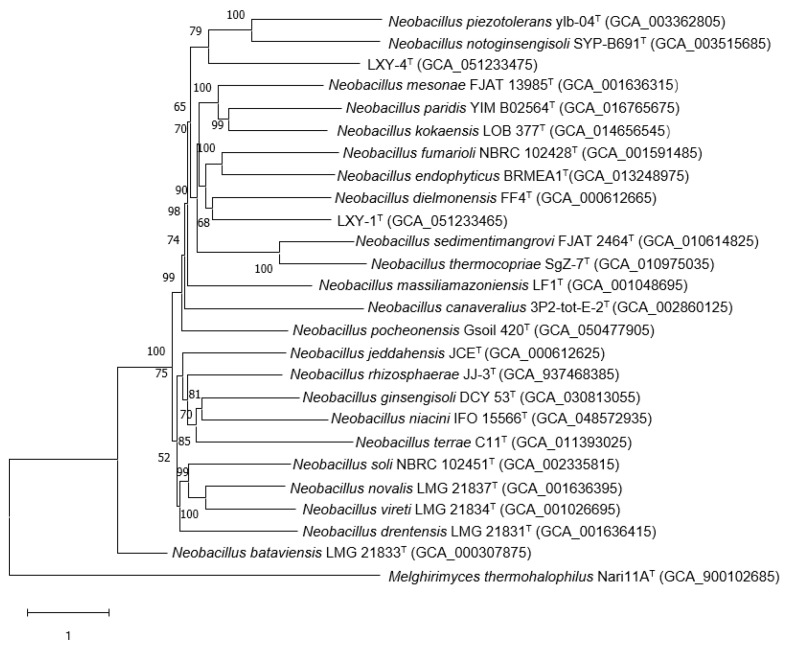
Phylogenomic tree of strains LXY-1^T^ and LXY-4^T^ within the members of genus *Neobacillus*. Phylogenetic relationships at the genomic level of strains LXY-1^T^ and LXY-4^T^ and the species of the genus *Neobacillus*. GenBank accession numbers of the genomes used are given in parentheses. The gene support indices indicate the number of single gene trees supporting each branch in the tree from the concatenated alignment and are marked on the branches. *Melghirimyces thermohalophilus* Nari11A^T^ was used as the out-group. Bars, 1 substitution per site.

Phylogenomic reconstruction based on IPGA [25] (https://nmdc.cn/ipga/, accessed on 2 September 2025) robustly placed strains LXY-1^T^ and LXY-4^T^ within the genus *Neobacillus*, with each strain forming a well-supported monophyletic clade (Figure 4). Notably, strain LXY-1^T^ showed relatedness to *Neobacillus endophyticus* BRMEA1^T^ (dDDH 20.70%, ANI 73.75%) and *Neobacillus fumarioli* NBRC 102428^T^ (dDDH 19.10%, ANI 73.17%) but formed a distinct subclade with *Neobacillus dielmonensis* FF4^T^ (dDDH 19.10%, ANI 72.84%), highlighting potential taxonomic complexity within *Neobacillus*. In contrast, strain LXY-4^T^ clustered distantly from its closest described relatives, with digital DNA–DNA hybridization (dDDH) and average nucleotide identity (ANI) values significantly below species thresholds (vs. *Cytobacillus oceanisediminis* CGMCC 1.10115^T^: dDDH 20.80%, ANI 69.76%; vs. *Neobacillus canaveralius* M4.6^T^: dDDH 20.40%, ANI 69.88%; vs. *Mesobacillus harenae* Y40^T^: dDDH 20.60%, ANI 69.12%; vs. *Mesobacillus jeotgali* DSM 18226^T^: dDDH 22.00%, ANI 69.06%; vs. *Mesobacillus subterraneus* DSM 13966^T^: dDDH 20.30%, ANI 68.72%).

The whole-genome sequence of LXY-1^T^ (GenBank accession no. JBKFGJ000000000) consisted of 4.54 Mb with a GC content of 38.61 mol%, whereas that of LXY-4^T^ (GenBank accession no. JBKFGH000000000) was 4.41 Mb with a GC content of 38.75 mol%. The average nucleotide identity (ANI) values of LXY-1^T^ were 78.74%, while the values of LXY-4^T^ were 71.09%, respectively, which confirmed their classification as distinct species. Based on the KEGG annotation, the genome of LXY-1^T^ was found to encode 128 genes associated with cellular processes, 2629 genes related to metabolism, 207 genes involved in genetic information processing, 65 genes linked to organismal systems, and 194 genes involved in environmental information processing. In comparison, LXY-4^T^ harbored 229 genes related to cellular processes, 4674 genes associated with metabolism, 262 genes involved in genetic information processing, 93 genes linked to organismal systems, and 335 genes involved in environmental information processing (Table S4).

**Figure 5:**
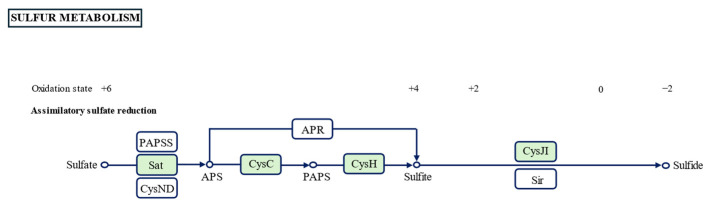
Partial sulfur metabolism pathway annotated in the KEGG database (ko00920). Genes in LXY-1^T^ and LXY-4^T^ were marked green.

These genomic findings were corroborated by phenotypic assays, including GEN III MicroPlate (Biolog), API ZYM, and API 20 NE systems, which demonstrated consistent metabolic profiles. Notably, different from other strains in the genus *Neobacillus*, genomic annotation revealed that strains LXY-1^T^ and LXY-4^T^, unlike other members of the genus *Neobacillus*, possess KEGG-annotated genes involved in sulfur cycle processes (Figure 5). These genetic determinants confer the capacity to convert sulfate (SO_4_^2−^) to hydrogen sulfide (H_2_S), a finding corroborated by phenotypic evidence from lead acetate test strips that visually confirmed H_2_S production. This concordance between genomic predictions and phenotypic manifestations validates the functional expression of sulfur metabolism pathways in these novel isolates. The observed sulfur-transforming capacity aligns with a broader biogeochemical context where the sulfate reduction pathway (SRP), mediated by taxonomically diverse microbial consortia [43], mediates sulfur reduction and precipitation. Beyond its roles in organic matter degradation and energy metabolism, this pathway plays a pivotal role in heavy metal environments: sulfate-reducing bacteria (SRB) generate hydrogen sulfide (H_2_S) through sulfate reduction [44], which reacts with heavy metals (e.g., Pb^2+^, Cd^2+^) to form insoluble metal sulfide precipitates, enabling biogenic immobilization and remediation of heavy metal pollution. This biogenic precipitation mechanism not only effectively immobilizes toxic metals through mineral encapsulation but also reduces their bioavailability, thereby enabling in situ bioremediation of polluted ecosystems. The coupling of sulfur cycling with heavy metal sequestration exemplifies microbially driven geochemical processes that bridge elemental cycles, offering a sustainable alternative to conventional physicochemical remediation approaches while advancing our understanding of ecosystem services in contaminated environments.

**Figure 6:**
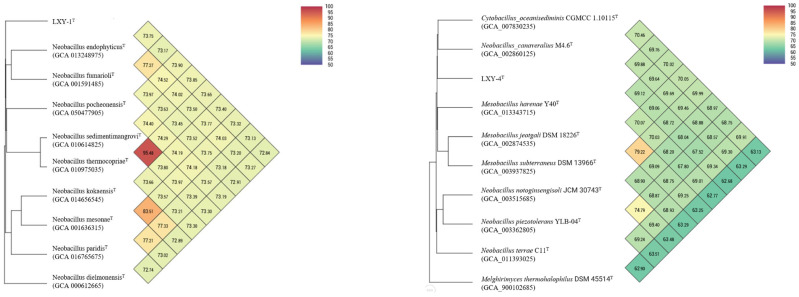
ANI heatmap of strains LXY-1^T^ and LXY-4^T^, along with their closely related strains. Heatmap of ANI values between strains LXY-1^T^ and LXY-4^T^ and their closely related strains.

As demonstrated by our comprehensive genomic analyses, the two novel strains LXY-1^T^ and LXY-4^T^ exhibit taxonomic and functional characteristics that support their classification as distinct species within the genus *Neobacillus*. Their genomic DNA G+C contents (38.61 mol% and 38.75 mol%) are consistent with the established range for the genus [1]. Furthermore, phylogenomic and comparative genomic analyses provide robust evidence for their novel taxonomic status. Specifically, these strains exhibit dDDH values (<20.40%) and ANI values (<78.74%) that are markedly below the recognized species delineation thresholds (dDDH <70%, ANI <95%) when compared with their closest type strains relatives (Figure 6). These molecular boundaries, together with their distinct genomic features, collectively fulfill the polyphasic criteria required for the designation of novel species within the genus *Neobacillus*.

### 3.4. Chemotaxonomic Characterization

When comparing the fatty acid profiles of strains LXY-1^T^ and LXY-4^T^ with those of their closest phylogenetic relatives, namely *Neobacillus drentensis* LMG 21831^T^ and *Neobacillus bataviensis* LMG 21833^T^, it was found that they exhibited both conserved and distinct characteristics (Table 2). Both LXY-1^T^ and LXY-4^T^ strains demonstrated the typical fatty acid composition characteristic of the genus *Neobacillus*. Their fatty acid profiles were predominantly composed of C_16:0_, iso-C_15:0_, antéiso-C_15:0_, and antéiso-C_17:0_. Through comparative analysis, notable differences emerged. Strain LXY-1^T^ contained higher levels of C_16:0_, antéiso-C_15:0_, and antéiso-C_17:0_ compared with *Neobacillus drentensis* LMG 21831^T^. Conversely, the content of iso-C_15:0_ in LXY-1^T^ was lower. As for strain LXY-4^T^, it exhibited more pronounced deviations in its fatty acid profile from its reference strain, *Neobacillus bataviensis* LMG 21833^T^, which included fatty acids such as C_14:1_ ω5*c*, C_18:1_ ω9*c*, and C_18:0_. Specifically, there was a significant difference in the relative abundance of C_14:1_ ω5*c* (Table 2). The conservation of major fatty acids, including C_16:0_, iso-C_15:0_, antéiso-C_15:0_, and antéiso-C_17:0_, along with the distinct differences in the relative abundance of key components, offers robust evidence. This evidence strongly supports the classification of LXY-1^T^ and LXY-4^T^ as novel species within the genus *Neobacillus*.

Polar lipid distributions provided additional taxonomic discrimination (Figure 1E,F), while LXY-1^T^ and LXY-4^T^ maintained the fundamental *Neobacillus* lipid architecture (diphosphatidylglycerol (DPG), phosphatidylethanolamine (PE), and phosphatidylglycerol (PG)) shared with *Neobacillus drentensis* LMG 21831^T^ and *Neobacillus bataviensis* LMG 21833^T^. In addition, LXY-1^T^ was positive for unidentified aminolipids (AL1-2), unidentified aminophospholipids (APL1-4), and unidentified phospholipids (PL1); LXY-4^T^ was positive for unidentified aminolipids (AL1-3), unidentified aminophospholipids (APL1-4), unidentified phospholipids (PL1-3), and lipids (L1-2).

## 4. Conclusions

Based on phylogenetic and genomic characteristics, such as phylogenetic position, a distinct branch within the genus *Neobacillus* has been established, with 16S rRNA gene sequence similarity below 98.7%, ANI values less than 95%, and dDDH values under 70%. Furthermore, physiological and chemotaxonomic analyses indicate that strains LXY-1^T^ and LXY-4^T^ exhibit characteristics consistent with other members of the genus. These include the ability to produce acid from various carbon sources, oxidize ferrous ions, and tolerate specific pH and temperature ranges for growth. Taken together, these findings support the proposal that strains LXY-1^T^ and LXY-4^T^ represent two novel species within the genus *Neobacillus*, for which the names *Neobacillus terrisolis* sp. nov. and *Neobacillus solisequens* sp. nov. are proposed, respectively.

### 4.1. Description of Neobacillus terrisolis sp. nov.

*Neobacillus terrisolis* sp. nov. (ter.ri.so’lis. L. n. *terra*, soil; L. n. *solum*, soil; N.L. masc. adj. *terrisolis*, soil-dwelling, referring to the fact that the type strain of this new species was isolated from soil samples. This name highlights the ecological niche and origin of the bacterium, distinguishing it from other species within the *Neobacillus* genus that may have different habitats or isolation sources.)

Cells are Gram-positive, facultatively anaerobic, spore-forming, non-motile, and short-oval shaped. After one day of aerobic incubation on R2A medium at 30 °C, colonies are 1–2 mm in diameter, round, creamy-white, and opaque. The optimal growth conditions are as follows: temperature, 22 °C; pH, 10.0; and 0% (*w*/*v*) NaCl concentration. The major cellular fatty acids comprise C_14:0_, C_16:0_, iso-C_15:0_, antéiso-C_15:0_, and antéiso-C_17:0_. The polar lipids consist of diphosphatidylglycerol (DPG), phosphatidylethanolamine (PE), phosphatidylglycerol (PG), unidentified aminolipids (AL1-2), unidentified aminophospholipids (APL1-4), and unidentified phospholipid (PL1).

The genomic DNA G+C content of the type strain is 38.61 mol%. The type strain, LXY-1^T^ (=CGMCC 30313^T^ = JCM 37671^T^), was isolated from a soil sample collected from a heavy metal smelting plant in Guangxi, China. The 16S rRNA gene sequence has been deposited in the GenBank/EMBL/DDBJ databases under accession number PP800207. Additionally, the complete genome sequence has been submitted to DDBJ/ENA/GenBank under accession number JBKFGJ000000000 (version JBKFGJ000000000.1).

### 4.2. Description of Neobacillus solisequens sp. nov.

*Neobacillus solisequens* sp. nov. (so.li.se’quens. L. neut. n. *solum*, soil; L. part. *sequens*, indicating a close connection or association; N.L. masc. adj. *solisequens*, soil-associated, referring to the characteristic of the type strain of this new species, which was isolated from soil and demonstrates physiological and ecological features closely tied to the soil habitat, such as its ability to utilize certain soil-derived nutrients and adapt to the specific microenvironmental conditions prevalent in soil).

Cells are Gram-positive, spore-forming, approximately 1 µm in width and 3 µm in length. Facultatively anaerobic, non-motile, and rod-shaped. After two days of incubation on R2A medium at 30 °C, colonies are 0.5–1 mm in diameter, circular, beige, and slightly translucent. The optimal growth conditions are as follows: temperature, 22 °C; pH, 10.0; and 0% (*w*/*v*) NaCl concentration. The major cellular fatty acids contain C_14:1_ ω5*c*, C_16:0_, iso-C_15:0_, iso-C_17:0_, antéiso-C_15:0_, and antéiso-C_17:0_. The polar lipids consist of diphosphatidylglycerol (DPG), phosphatidylethanolamine (PE), phosphatidylglycerol (PG), unidentified aminolipids (AL1-3), unidentified aminophospholipids (APL1-4), unidentified phospholipids (PL1-3), and lipids (L1-2).

The genomic DNA G+C content of the type strain is 38.75 mol%. The type strain, LXY-4^T^ (=CGMCC 1.62901^T^ = JCM 37672^T^), was isolated from a soil sample collected from a heavy metal smelting plant in Guangxi, China. The 16S rRNA gene sequence has been deposited in the GenBank/EMBL/DDBJ databases under accession number PQ578319. Additionally, the complete genome sequence has been submitted to DDBJ/ENA/GenBank under accession number JBKFGH000000000 (version JBKFGH000000000.1).

## Figures and Tables

**Table 1:** Differential characteristics of strains LXY-1^T^ and LXY-4^T^ and closely related type strains within the genus *Neobacillus*. Strains: 1, LXY-1^T^ (data from this study); 2, LXY-4^T^ (data from this study); 3, *N. drentensis* LMG 21831^T^ (data from reference [41]); 4, *N. bataviensis* LMG 21833^T^ (data from reference [41]); +, positive; −, negative; v, results vary between strains; *, data from the present study.

Characteristics	1	2	3	4
Source of Isolation	Soil	Soil	Soil	Soil
Cell shape	Short-oval	Rod-shaped	Tapered rod-shaped	Slightly tapered rod-shaped
Anaerobic growth	+	+	+	+
Temperature (°C) Optimal (°C)	22–50 (22)	22–50 (22)	Up to 50–55 (30)	Up to 50–55 (30)
pH	6.0–12.0	7.0–12.0	5.5–10.0	4.0–10.0
Gram staining	+	+	+	+
Major cellular fatty acids	Antéiso C_15:0_	Antéiso C_15:0_	Antéiso C_15:0_, iso-C_15:0_ and C_16:1_ ω11*c*	Antéiso C_15:0_, iso-C_15:0_ and C_16:1_ ω11*c*
*β*-Galactosidase (ONPG)	−	−	v	+
Nitrate reduction to N_2_	−	+	v	+
**Genome features**				
16S rRNA gene identities *	97.3	96.7	95.7	100
Mol% G+C	38.61	38.75	39	39.5
ANI *	78.74	71.09	77.04	79.41
**Acid production from**				
L-Fucose	−	−	−	−
Lactose	−	−	−	−
D-Mannitol	−	−	−	−
Melibiose	−	−	+	−
Raffinose	−	−	v	−
Salicin	−	+	+	−
Sucrose	−	+	v	−
Turanose	−	−	v	−

**Table 2:** Cellular fatty acid profiles (% of totals) of strains LXY-1^T^, LXY-4^T^, data from this study; *N. drentensis* LMG 21831^T^ and *N. bataviensis* LMG 21833^T^, data from reference [41]. Data are mean percentages of total fatty acids ± SD. Only fatty acids accounting for at least 1.0% of the total fatty acid content are listed.

Fatty Acid	Percentage (%)			
	LXY-1^T^	LXY-4^T^	*N. drentensis* LMG 21831^T^	*N. bataviensis* LMG 21833^T^
iso-C_14:0_	-	-	8.7 ± 2.5	6.9 ± 1.1
C_14:0_	1.94	-	1.4 ± 1.3	1.5 ± 0.6
C_16:1_ *ω*7*c* alcohol	-	-	3.1 ± 0.8	2.3 ± 0.8
C_14:1_ *ω*5*c*	-	7.97	-	-
C_16:0_	11.94	5.49	3.4 ± 1.6	7.7 ± 3.2
iso-C_15:0_	14.61	13.93	32.2 ± 4.2	36.9 ± 4.3
antéiso-C_15:0_	44.14	33.52	21.8 ± 5.6	20.5 ± 3.0
iso-C_17:0_	-	11.43	2.6 ± 0.6	1.4 ± 0.8
antéiso-C_17:0_	12.92	17.28	1.1 ± 0.3	11.1 ± 0.4
C_18:1_ *ω*9*c*	-	-	1.8 ± 1.1	1.3 ± 0.8
C_18:0_	-	-	1.3 ± 0.9	2.7 ± 1.4

## Data Availability

The original contributions presented in this study are included in the article/Supplementary Materials. Further inquiries can be directed to the corresponding authors.

## Supplementary Materials

The following supporting information can be downloaded at https://www.mdpi.com/article/10.3390/microorganisms13112437/s1, Figure S1: Maximum-likelihood tree based on 16S rRNA gene sequences showing the phylogenetic position of strains LXY-1^T^ and LXY-4^T^ among the members of genus *Neobacillus*. *Melghirimyces thermohalophilus* Nari11A^T^ served as the out-group. Figure S2: Maximum-parsimony tree based on 16S rRNA gene sequences showing the phylogenetic position of strains LXY-1^T^ and LXY-4^T^ among the members of genus *Neobacillus*. *Melghirimyces thermohalophilus* Nari11A^T^ served as the out-group. Table S1: Phenotypic characteristics of strains LXY-1^T^ and LXY-4^T^ determined by Biolog GEN III MicroPlate^TM^. Table S2: Phenotypic characteristics of strains LXY-1^T^ and LXY-4^T^ determined by the API ZYM kit. Table S3: Phenotypic characteristics of strains LXY-1^T^ and LXY-4^T^ determined by the API 20NE test kit. Table S4: Phenotypic characteristics of strains LXY-1^T^ and LXY-4^T^ determined.

## Abbreviations

The following abbreviations are used in this manuscript:

ANI	Average nucleotide identity
dDDH	Digital DNA–DNA hybridization
ML	Maximum-likelihood
MP	Maximum parsimony
NJ	Neighbor-joining
R2A	Reasoner’s 2A

## Appendix A

The 16S rRNA gene sequences of strains *Neobacillus terrisolis* sp. nov. and *Neobacillus solisequens* sp. nov. are PP800207 and PQ578319, respectively. The whole-genome shotgun projects for *Neobacillus terrisolis* sp. nov. and *Neobacillus solisequens* sp. nov. have been deposited at DDBJ/ENA/GenBank under the accession numbers JBKFGJ000000000 and JBKFGH000000000, respectively. The respective versions described in this paper are JBKFGJ000000000.1 and JBKFGH000000000.1.

## References

[1] Patel S., Gupta R.S. (2020). A Phylogenomic and Comparative Genomic Framework for Resolving the Polyphyly of the Genus *Bacillus*: Proposal for Six New Genera of *Bacillus* Species, *Peribacillus* gen. nov., *Cytobacillus* gen. nov., *Mesobacillus* gen. nov., *Neobacillus* gen. nov., *Metabacillus* gen. nov. And *Alkalihalobacillus* gen. nov. Int. J. Syst. Evol. Microbiol..

[2] Jiang L.M., Lee M.H., Jeong J.C., Kim D.H., Kim C.Y., Kim S.W., Lee J. (2021). *Neobacillus endophyticus* sp. nov., an Endophytic Bacterium Isolated from Selaginella Involvens Roots. Int. J. Syst. Evol. Microbiol..

[3] Zhan P.C., Li C.J., Zhang Z., Mao R.F., Liu J.R., Jiang X.W., Zhi X.Y., Yang L.L. (2022). *Neobacillus paridis* sp. nov., an Endophyte of Paris Polyphylla Smith Var. Yunnanensis. Arch. Microbiol..

[4] Han D.M., Choi D.G., Baek J.H., Hao L.J., Jeon C.O. (2023). *Neobacillus terrae* sp. nov., Isolated from Mountain Soil. Int. J. Syst. Evol. Microbiol..

[5] Yuki K., Matsubara H., Yamaguchi S. (2022). *Neobacillus kokaensis* sp. nov., Isolated from Soil. Int. J. Syst. Evol. Microbiol..

[6] Ten L.N., Baek S.H., Im W.T., Liu Q.M., Aslam Z., Lee S.T. (2006). *Bacillus panaciterrae* sp. nov., Isolated from Soil of a Ginseng Field. Int. J. Syst. Evol. Microbiol..

[7] Caccamo D., Gugliandolo C., Stackebrandt E., Maugeri T.L. (2000). *Bacillus vulcani* sp. nov., a Novel Thermophilic Species Isolated from a Shallow Marine Hydrothermal Vent. Int. J. Syst. Evol. Microbiol..

[8] Liu B., Liu G.H., Hu G.H., Chen M.C. (2014). *Bacillus mesonae* sp. nov., Isolated from the Root of Mesona Chinensis. Int. J. Syst. Evol. Microbiol..

[9] Kämpfer P., Busse H.J., Glaeser S.P., Kloepper J.W., Hu C.H., McInroy J.A. (2016). *Bacillus cucumis* sp. nov Isolated from the Rhizosphere of Cucumber (*Cucumis sativus*). Int. J. Syst. Evol. Microbiol..

[10] Kaempfer P., Glaeser S.P., McInroy J.A., Clermont D., Lipski A., Criscuolo A. (2022). *Neobacillus rhizosphaerae* sp. nov., Isolated from the Rhizosphere, and Reclassification of *Bacillus dielmonensis* as *Neobacillus dielmonensis* comb. nov. Int. J. Syst. Evol. Microbiol..

[11] Han L.C., Yang G.Q., Zhou X.M., Yang D.H., Hu P., Lu Q., Zhou S.G. (2013). *Bacillus thermocopriae* sp. nov., Isolated from a Compost. Int. J. Syst. Evol. Microbiol..

[12] Yu L.B., Tang X.X., Wei S.P., Qiu Y.K., Xu X.S.T., Xu G.X., Wang Q.L., Yang Q. (2019). Two Novel Species of the Family Bacillaceae: *Oceanobacillus piezotolerans* sp. nov. And *Bacillus piezotolerans* sp. nov., from Deep-Sea Sediment Samples of Yap Trench. Int. J. Syst. Evol. Microbiol..

[13] Bittar F., Bibi F., Ramasamy D., Lagier J.C., Azhar E.I., Jiman-Fatani A.A., Al-Ghamdi A.K., Nguyen T.T., Yasir M., Fournier P.E. (2015). Non Contiguous-Finished Genome Sequence and Description of *Bacillus jeddahensis* sp. nov. Stand. Genom. Sci..

[14] El-Sapagh S.H., El-Zawawy N.A., Elshobary M.E., Alquraishi M., Zabed H.M., Nouh H.S. (2024). Harnessing the Power of *Neobacillus niacini* Aumc-B524 for Silver Oxide Nanoparticle Synthesis: Optimization, Characterization, and Bioactivity Exploration. Microb. Cell Factories.

[15] Tan Z., Guo X., Tan X., Liang Q., Peng G. (2024). New Neobacillus mesonae Fjat-13985ndj36 Strain Is Preserved in Guangdong Microbiological Culture Collection Center, Used for Preventing and Treating Rice Sheath Blight and Plant Blight by Inhibiting Rhizoctonia solani, and Fusarium oxysporum and Producing Protease.

[16] Ryu H.W., Yoo S.K., Choi J.M., Cho K.S., Cha D.K. (2009). Thermophilic Biofiltration of H_2_S and Isolation of a Thermophilic and Heterotrophic H_2_S-Degrading Bacterium, *Bacillus* Sp Tso3. J. Hazard. Mater..

[17] Tyulenev A., Smirnova G., Ushakov V., Kalashnikova T., Sutormina L., Oktyabrsky O. (2024). Stress-Induced Sulfide Production by *Bacillus subtilis* and *Bacillus megaterium*. Microorganisms.

[18] Tang C., Li J.J., Shen Y.M., Liu M.H., Liu H.L., Liu H.W., Xun L.Y., Xia Y.Z. (2023). A Sulfide-Sensor and a Sulfane Sulfur-Sensor Collectively Regulate Sulfur-Oxidation for Feather Degradation by *Bacillus licheniformis*. Commun. Biol..

[19] Goyal P., Belapurkar P., Kar A. (2019). A Review on in Vitro and in Vivo Bioremediation Potential of Environmental and Probiotic Species of *Bacillus* and Other Probiotic Microorganisms for Two Heavy Metals, Cadmium and Nickel. Biosci. Biotechnol. Res. Asia.

[20] Belapurkar P., Goyal P., Kar A. (2016). In Vitro Evaluation of Bioremediation Capacity of a Commercial Probiotic, *Bacillus coagulans*, for Chromium (Vi) and Lead (Ii) Toxicity. J. Pharm. Bioallied Sci..

[21] Lane D.J., Pace B., Olsen G.J., Stahl D.A., Sogin M.L., Pace N.R. (1985). Rapid-Determination of 16s Ribosomal-Rna Sequences for Phylogenetic Analyses. Proc. Natl. Acad. Sci. USA.

[22] Reasoner D.J., Geldreich E.E. (1985). A New Medium for the Enumeration and Subculture of Bacteria from Potable Water. Appl. Environ. Microbiol..

[23] Weisburg W.G., Barns S.M., Pelletier D.A., Lane D.J. (1991). 16s Ribosomal DNA Amplification for Phylogenetic Study. J. Bacteriol..

[24] Stackebrandt E., Goebel B.M. (1994). A Place for DNA-DNA Reassociation and 16s Ribosomal-Rna Sequence-Analysis in the Present Species Definition in Bacteriology. Int. J. Syst. Bacteriol..

[25] Liu D.M., Zhang Y.F., Fan G.M., Sun D.Z., Zhang X.J., Yu Z.F., Wang J.F., Wu L.H., Shi W.Y., Ma J.C. (2022). Ipga: A Handy Integrated Prokaryotes Genome and Pan-Genome Analysis Web Service. iMeta.

[26] Hucker G.J. (1921). A New Modification and Application of the Gram Stain. J. Bacteriol..

[27] Preston-Mafham J., Boddy L., Randerson P.F. (2002). Analysis of Microbial Community Functional Diversity Using Sole-Carbon-Source Utilisation Profiles—A Critique. FEMS Microbiol. Ecol..

[28] Wilkins T.D., Abramson I.J., Moore W.E.C., Holdeman L.V. (1972). Standardized Single-Disk Method for Antibiotic Susceptibility Testing of Anaerobic Bacteria. Antimicrob. Agents Chemother..

[29] Wang Y.J., Xu X.J., Zhou N., Sun Y.T., Liu C., Liu S.J., You X. (2019). *Parabacteroides acidifaciens* sp. nov., Isolated from Human Faeces. Int. J. Syst. Evol. Microbiol..

[30] Abdugheni R., Li D.H., Wang Y.J., Du M.X., Zhou N., Liu C., Liu S.J. (2023). *Acidaminococcus homini s* sp. nov., *Amedibacillus hominis* sp. nov., *Lientehia hominis* gen. nov. sp. nov., *Merdimmobilis hominis* gen. nov. sp. nov., and *Paraeggerthella hominis* sp. nov., Isolated from Human Faeces. Int. J. Syst. Evol. Microbiol..

[31] Schoch C.L., Ciufo S., Domrachev M., Hotton C.L., Kannan S., Khovanskaya R., Leipe D., McVeigh R., O’Neill K., Robbertse B. (2020). Ncbi Taxonomy: A Comprehensive Update on Curation, Resources and Tools. Database.

[32] Saitou N., Nei M. (1987). The Neighbor-Joining Method—A New Method for Reconstructing Phylogenetic Trees. Mol. Biol. Evol..

[33] Kumar S., Stecher G., Li M., Knyaz C., Tamura K. (2018). Mega X: Molecular Evolutionary Genetics Analysis across Computing Platforms. Mol. Biol. Evol..

[34] Felsenstein J. (1981). Evolutionary Trees from DNA-Sequences—A Maximum-Likelihood Approach. J. Mol. Evol..

[35] Yan Z., Cao Z., Liu Y.S., Ogilvie H.A., Nakhleh L. (2022). Maximum Parsimony Inference of Phylogenetic Networks in the Presence of Polyploid Complexes. Syst. Biol..

[36] Kumar S., Stecher G., Tamura K. (2016). Mega7: Molecular Evolutionary Genetics Analysis Version 7.0 for Bigger Datasets. Mol. Biol. Evol..

[37] Abdugheni R., Liu C., Liu F.L., Zhou N., Jiang C.Y., Liu Y.H., Li L., Li W.J., Liu S.J. (2023). Comparative Genomics Reveals Extensive Intra-Species Genetic Divergence of the Prevalent Gut Commensal *Ruminococcus gnavu*. Microb. Genom..

[38] Parks D.H., Imelfort M., Skennerton C.T., Hugenholtz P., Tyson G.W. (2015). Checkm: Assessing the Quality of Microbial Genomes Recovered from Isolates, Single Cells, and Metagenomes. Genome Res..

[39] Bankevich A., Nurk S., Antipov D., Gurevich A.A., Dvorkin M., Kulikov A.S., Lesin V.M., Nikolenko S.I., Pham S., Prjibelski A.D. (2012). Spades: A New Genome Assembly Algorithm and Its Applications to Single-Cell Sequencing. J. Comput. Biol..

[40] Meier-Kolthoff J.P., Göker M. (2019). Tygs Is an Automated High-Throughput Platform for State-of-the-Art Genome-Based Taxonomy. Nat. Commun..

[41] Heyrman J., Vanparys B., Logan N.A., Balcaen A., Rodíguez-Díaz M., Felske A., De Vos P. (2004). *Bacillus novalis* sp. nov., *Bacillus vireti* sp. nov., *Bacillus soli* sp. nov., *Bacillus bataviensis* sp. nov. and *Bacillus drentensis* sp. nov., from the Drentse a Grasslands. Int. J. Syst. Evol. Microbiol..

[42] Kanso S., Greene A.C., Patel B.K.C. (2002). *Bacillus subterraneus* sp. nov., an Iron- and Manganese-Reducing Bacterium from a Deep Subsurface Australian Thermal Aquifer. Int. J. Syst. Evol. Microbiol..

[43] Gittel A., Seidel M., Kuever J., Galushko A.S., Cypionka H., Könneke M. (2010). *Desulfopila inferna* sp nov., a Sulfate-Reducing Bacterium Isolated from the Subsurface of a Tidal Sand-Flat. Int. J. Syst. Evol. Microbiol..

[44] Hao O.J., Chen J.M., Huang L., Buglass R.L. (1996). Sulfate-Reducing Bacteria. Crit. Rev. Environ. Sci. Technol..

