# Supplementary material for: Neobacillus terrisolis sp. nov. and Neobacillus solisequens sp. nov. Isolated from Soil
Source: Microorganisms. 2025 Oct 24;13(11):2437. doi: 10.3390/microorganisms13112437 (PMC12654729; doi:10.3390/microorganisms13112437)
Supplement: Supplementary file 1 [file microorganisms-13-02437-s001.zip › microorganisms-3923284-supplementary.pdf]

Supplementary

Neobacillus terrisolis sp. nov. and *Neobacillus solisequens* sp. nov. isolated from soil

**Haoyu Wu [1,2], Congguo Ran [2,3], Nan Zhou [2], Xize Zhao [2,3], Xingyu Liu [1,*], Chengying Jiang [2,3,*], Yinghao Zhao [1]
and Ying Lv [1]**

[1] State Key Laboratory of Geological Processes and Mineral Resources, China University of Geosciences, Beijing 100083, China
[2] State Key Laboratory of Microbial Diversity and Innovative Utilization, Environmental Microbiology Research Center, Institute of Microbiology, Chinese Academy of Sciences, Beijing 100101, China
[3] University of Chinese Academy of Sciences, Beijing 100049, China
* Correspondence: wellwoodliu@163.com (X.L.); jiangcy@im.ac.cn (C.J.)

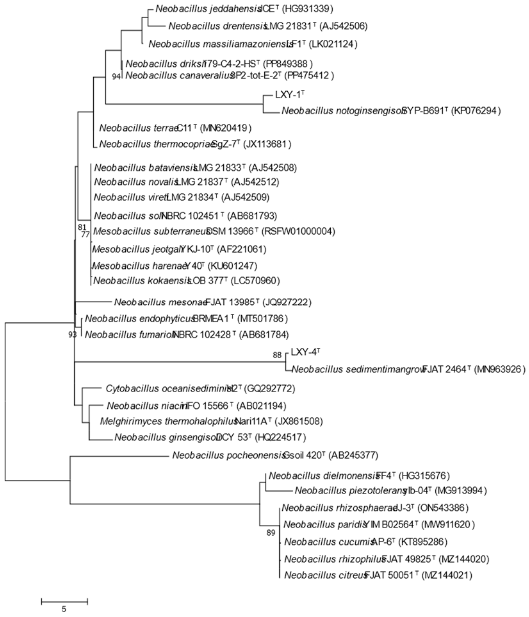

Figure S1. Maximum-likelihood tree based on 16S rRNA gene sequences showing the phylogenetic position of strains LXY-1^{T} and LXY-4^{T} among the members of genus *Neobacillus*. *Melghirimyces thermohalophilus* Nari11A^{T} served as the out-group. The sequences were obtained from GenBank. Bar, 5 substitutions per nucleotide position.

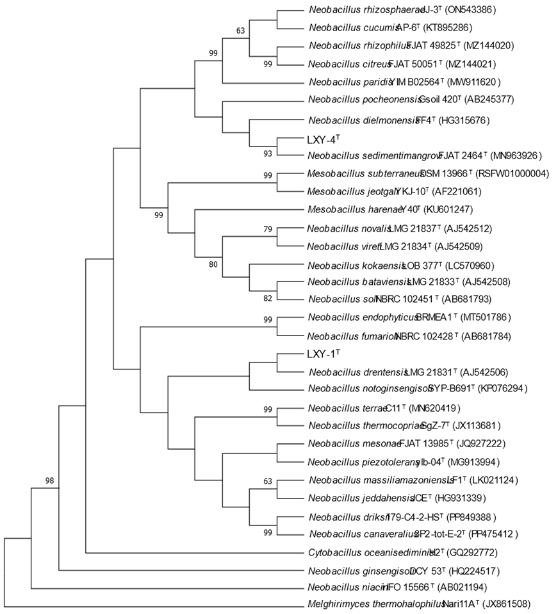

Figure S2. Maximum-parsimony tree based on 16S rRNA gene sequences showing the phylogenetic position of strains LXY-1^{T} and LXY-4^{T} among the members of genus *Neobacillus*. *Melghirimyces thermohalophilus* Nari11A^{T} served as the out-group. The sequences were obtained from GenBank.

Table S1. Phenotypic characteristics of strains LXY-1^{T} and LXY-4^{T} determined by Biolog GEN III MicroPlateTM. "+": Positive; "-": Negative

NO.	Characteristic	LXY-1^{T}	LXY-4^{T}

	Carbon source utilization assay		
A1	Negative control	−	−
A2	Dextrin	+	−
A3	D-Maltose	−	−
A4	D-Trehalose	−	−
A5	D-Cellobiose	−	−
A6	Gentiobiose	−	+
A7	Sucrose	−	+
A8	D-Turanose	−	−
A9	Stachyose	−	−
B1	D-Raffinose	−	−
B2	α-D-Lactose	−	−
B3	D-Melibiose	−	−
B4	β-Methyl-D-Glucoside	−	+
B5	D-Salicin	−	+
B6	N-Acetyl-D-Glucosamine	+	+
B7	N-Acetyl-β-D-Mannosamine	−	−
B8	N-Acetyl-D-Galactosamine	−	−
B9	N-Acetyl-Neuraminic acid	−	−
C1	α-D-Glucose	+	+
C2	D-Mannose	−	−
C3	D-Fructose	+	+
C4	D-Galactose	−	−
C5	3-Methyl glucose	−	−
C6	D-Fucose	−	−
C7	L-Fucose	−	−
C8	L-Rhamnose	−	−
C9	Inosine	−	+
D1	D-Sorbitol	−	+
D2	D-Mannitol	−	+
D3	D-Arabitol	−	+
D4	myo-Inositol	−	−
D5	Glycerol	+	+
D6	D-Glucose-6-PO4	+	−
D7	D-Fructose-6-PO4	+	−
D8	D-Aspartic Acid	−	−
D9	D-Serine	+	−
E1	Gelatin	+	−
E2	Glycyl-L-Proline	−	−
E3	L-Alanine	+	−
E4	L-Arginine	−	−
E5	L-Aspartic acid	−	−
E6	L-Glutamic acid	−	−
E7	L-Histidine	−	−

E8	L-Pyroglutamic acid	−	−
E9	L-Serine	+	−
F1	Pectin	+	+
F2	D-Galacturonic acid	−	−
F3	L-Galactonic acid lactone	−	−
F4	D-Gluconic acid	+	−
F5	D-Glucuronic acid	−	−
F6	Glucuronamide	−	−
F7	Mucic acid	−	−
F8	Quinic acid	−	−
F9	D-Saccharic acid	−	+
G1	p-Hydroxy-Phenylacetic acid	−	−
G2	Methyl pyruvate	+	−
G3	D-Lactic acid methyl ester	−	+
G4	L-Lactic acid	+	+
G5	Citric acid	−	−
G6	α-Keto-Glutaric acid	−	−
G7	D-Malic acid	−	−
G8	L-Malic acid	+	+
G9	Bromo-Succinic acid	+	−
H1	Tween 40	−	−
H2	γ-Amino-Butyric acid	−	−
H3	α-Hydroxy-Butyric acid	−	+
H4	β-Hydroxy-D,L-Butyric acid	−	−
H5	α-Keto-Butyric acid	−	−
H6	Acetoacetic acid	−	−
H7	Propionic acid	−	−
H8	Acetic acid	−	+
H9	Formic acid	+	−
	Chemical sensitivity assay		
A10	Positive control	+	+
A11	pH 6	+	+
A12	pH 5	−	−
B10	1% NaCl	+	+
B11	4% NaCl	+	−
B12	8% NaCl	+	−
C10	1% Sodium lactate	+	+
C11	Fusidic acid	−	−
C12	D-Serine	+	−
D10	Troleandomycin	−	−
D11	Rifamycin SV	+	−
D12	Minocycline	−	−
E10	Lincomycin	−	−
E11	Guanidine HCl	+	−

E12	Niaproof 4	−	−
F10	Vancomycin	−	−
F11	Tetrazolium violet	−	+
F12	Tetrazolium blue	−	+
G10	Nalidixic acid	−	+
G11	Lithium chloride	+	−
G12	Potassium tellurite	−	+
H10	Aztreonam	+	−
H11	Sodium butyrate	+	−
H12	Sodium bromate	+	−

Table S2. Phenotypic characteristics of strains LXY-1^T and LXY-4^T determined by the API ZYM kit. "+": Positive; "-": Negative

NO.	Characteristic	LXY-1^T	LXY-4^T
1	Control	−	−
2	Alkaline phosphatase	+	+
3	Esterase (C4)	+	−
4	Esterase (C8)	+	−
5	Lipase (C14)	−	−
6	Leucine aminopeptidase	+	−
7	Valine aminopeptidase	−	−
8	Cysteine aminopeptidase	−	−
9	Trypsin	+	+
10	Chymotrypsin	+	−
11	Acid phosphatase	+	+
12	Phosphoamidase	+	+
13	*α*-Galactosidase	−	−
14	*β*-Galactosidase	−	−
15	*β*-Glucuronidase	−	−
16	*α*-Glucosidase	+	−
17	*β*-Glucosidase	−	+
18	*N*-acetyl-*β*-glucosaminidase	−	−
19	α-mannosidase	−	−
20	α-fucosidase	−	−

Table S3. Phenotypic characteristics of strains LXY-1^T and LXY-4^T determined by the API 20NE test kit. "+": Positive; "-": Negative

NO.	Characteristic	LXY-1^T	LXY-4^T
1	Reduction of nitrates to nitrites	+	+
2	Reduction of nitrates to nitrogen	−	+
3	Indole production (tryptophan)	−	−

4	Fermentation (glucose)	−	−
5	Arginine dihydrolase	−	−
6	Urease	−	−
7	Hydrolysis (β-glucosidase) (esculin)	+	+
8	Hydrolysis (protease) (gelatin)	+	−
9	β-Galactosidase	−	−
10	Assimilation (glucose)	+	−
11	Assimilation (arabinose)	−	−
12	Assimilation (mannose)	−	−
13	Assimilation (mannitol)	+	+
14	Assimilation (N-acetyl-glucosamine)	+	+
15	Assimilation (maltose)	+	−
16	Assimilation (potassium gluconate)	+	+
17	Assimilation (capric acid)	−	−
18	Assimilation (adipic acid)	+	−
19	Assimilation (malate)	+	+
20	Assimilation (trisodium citrate)	+	−
21	Assimilation (phenylacetic acid)	−	−
22	Cytochrome oxidase	−	−

Table S4. Phenotypic characteristics of strains LXY-1^{T} and LXY-4^{T} determined.

**Strain**	**LXY-1^{T}**	**LXY-4^{T}**
**Celler Processes**	128	229
1 Cell motility	41	52
2 Cell growth and death	22	29
3 Cellular community-prokartyotes	58	129
4 Transport and catabolism	7	18
5 Cellular community-eukaryotes	0	1
**Metabolism**	2629	4674
1 Biosynthesis of other secondary metabolites	36	77
2 Global and overview maps	1461	2548
3 Xenobiotics biodegradation and metabolism	44	104
4 Glycan biosynthesis and metabolism	85	111
5 Lipid metabolism	56	116
6 Carbohydrate metabolism	255	536
7 Metabolism of cofactors and vitamins	157	271
8 Metabolism of other amino acids	49	83
9 Amino acid metabolism	245	410
10 Metabolism of terpenoids and polyketides	26	36
11 Nucleotide metabolism	78	110
12 Energy metabolism	137	272
**Genetic Information Processing**	207	262
1 Transcription	6	7
2 Translation	88	84
3 Replication and repair	53	93
4 Folding, sorting and degradation	61	78
**Organismal Systems**	65	93
1 Immune system	7	9
2 Digestive system	13	15
3 Endocrine system	24	33

4 Aging	8	12
5 Nervous system	4	9
6 Environmental adaptation	8	14
7 Circulatory system	1	1
**Human Diseases**	105	166
1 Endocrine and metabolic disease	10	13
2 Substance dependence	0	3
3 Infectious disease: bacterial	21	24
4 Cancer: overview	10	25
5 Neurodegenerative disease	15	23
6 Cardiovascular disease	12	17
7 Infectious disease: viral	4	3
8 Cancer: specific types	3	2
9 Infectious disease: parasitic	3	4
10 Drug resistance: antineoplastic	5	6
11 Drug resistance: antimicrobial	20	45
12 Immune disease	2	1
**Environmental Information Processing**	194	335
1 Signal transduction	85	179
2 Membrane transport	109	154
3 Signaling molecules and interaction	0	2
